# 15296 A scoping review of challenges to approve community-engaged research proposals and best practices when working with the Institutional Review Board

**DOI:** 10.1017/cts.2021.606

**Published:** 2021-03-30

**Authors:** Janet Pan, Deborah Onakomaiya, Holly Tan, Smiti Nadkarni, Timothy Roberts, Antoinette M. Schoenthaler, Nadia Islam

**Affiliations:** 1 Department of Population Health, NYU Grossman School of Medicine; 2 UCLA Fielding School of Public Health; 3 Health Sciences Library, NYU Grossman School of Medicine

## Abstract

ABSTRACT IMPACT: This work will contribute to the understanding of challenges and best practices for navigating the research review process for collaborative community-engaged research. OBJECTIVES/GOALS: The aim of this scoping review is to comprehensively identify the challenges that researchers, community partners, and the Institutional Review Board (IRB) face, in order to develop best practices to guide future community-engaged research (CEnR). METHODS/STUDY POPULATION: Community-engaged research (CEnR) encompasses all research practices in which traditional researchers collaborate with community partners to identify health disparities that affect the community. CEnR aims to empower communities and prevent exploitative research practices on vulnerable populations. Though many goals of CEnR align with that of the Institutional Review Board (IRB) to protect human research subjects from unethical harm, researchers and community members conducting CEnR are often met with challenges when getting research approval. The search strategy included all publications pertaining to challenges in IRB approval and process for studies in the spectrum in community-engaged research. Systematic searches in PubMed Central and PsycINFO were conducted. RESULTS/ANTICIPATED RESULTS: The search strategy produced 748 publications from peer-reviewed journals. We included 118 publications that met our initial inclusion/exclusion criteria from the search strategy in our analysis. Preliminary results show that common challenges include lack understanding of the duo role of community members as researchers and participants, informed consent language barriers, and lack of understanding community-based participatory research. Best practices when working with the IRB include fostering an environment for open communication with the IRB early in the research process, understanding timeline constraints from both researcher and community agencies and supporting the role of community members as research staff. DISCUSSION/SIGNIFICANCE OF FINDINGS: Community-engaged research efforts are advantageous in empowering and providing agency for community members to address important health concerns within their communities. To prevent the exploitation of vulnerable and underserved populations, more research should engage in collaborative community-based partnerships.

